# RNA-Seq Profiling of Circular RNAs During Development of Hindgut in Rat Embryos With Ethylenethiourea-Induced Anorectal Malformations

**DOI:** 10.3389/fgene.2021.605015

**Published:** 2021-04-13

**Authors:** Si Ying Li, Chen Yi Wang, Yun Xia Xiao, Xiao Bing Tang, Zheng Wei Yuan, Yu Zuo Bai

**Affiliations:** ^1^Department of Pediatric Surgery, Shengjing Hospital of China Medical University, Shenyang, China; ^2^The Key Laboratory of Health Ministry for Congenital Malformation, Shenyang, China

**Keywords:** anorectal malformation, high-throughput RNA sequencing, circRNA, circRNA-miRNA-mRNA network, competing endogenous RNA

## Abstract

Anorectal malformations (ARMs) are among the most common congenital terminal digestive tract malformations. Circular RNAs (circRNAs), a novel type of endogenous non-coding RNAs, play roles in the development of the digestive system; however, their contributions to the pathogenesis of ARMs are not well-established. In this study, we explored the mechanism underlying ethylenethiourea (ETU)-induced ARMs by profiling circRNA expression via RNA-seq and constructing a regulatory circRNA-miRNA-mRNA network. Nine pregnant rats were gavage-fed a single dose of 125 mg/kg 1% ETU (ARM group) on gestational day 10 (GD10), and another 9 pregnant rats received a similar dose of saline (normal group) as a control. Embryos were obtained by cesarean section on the key time-points of anorectal development (GD14, GD15, and GD16). Hindgut samples isolated from the fetuses were evaluated by high-throughput sequencing and differentially expressed circRNAs were validated by reverse transcription-quantitative polymerase chain reaction, agarose gel electrophoresis, and Sanger cloning and sequencing. A total of 18295 circRNAs were identified in the normal and ARM groups. Based on the 425 differentially expressed circRNAs (|Fc| > 2, p < 0.05), circRNA-miRNA and miRNA-mRNA pairs were predicted using miREAP, miRanda, and TargetScan. A total of 55 circRNAs (14 up- and 41 downregulated in the ARM group compared to the normal group) were predicted to bind to 195 miRNAs and 947 mRNAs. Competing endogenous RNA networks and a Kyoto Encyclopedia of Genes and Genomes analysis revealed that novel_circ_001042 had the greatest connectivity and was closely related to ARM-associated signaling pathways, such as the Wingless Type MMTV integration site family, mitogen-activated protein kinase, and transforming growth factor-β pathways. These results provide original insight into the roles of circRNAs in ARMs and provide a valuable resource for further analyses of molecular mechanisms and signaling networks.

## Introduction

Anorectal malformations (ARMs) are the most common congenital terminal digestive tract malformations, with an incidence of approximately 1 in 5,000 births and a slightly lower incidence in females than in males (Cuschieri and Eurocat Working Group, 2001; Wood and Levitt, 2018). The wide spectrum of ARM phenotypes includes stenotic anus, ectopic anus, rectourethral fistula, and rectovestibular fistula (Endo et al., 1999; Bai et al., 2004; Wijers et al., 2014). More than half of patients with ARM also suffer from congenital anomalies of the urogenital system, cardiovascular system, skeleton, and gastrointestinal tract (Stoll et al., 2007; Nah et al., 2012). Despite sophisticated surgical reconstruction operations, many patients continue to experience complications, such as fecal incontinence and constipation, which adversely affect their quality of life (Grano et al., 2013; Rintala, 2016). Recent studies have identified genes and genetic signaling pathways involved in the remarkable pathophysiology of ARMs; however, the specific etiology remains unclear (Draaken et al., 2012; Wijers et al., 2014). Therefore, it is essential to investigate the pathogenesis of ARMs.

Circular RNAs (circRNAs) are a novel type of endogenous non-coding RNA that predominately exist in the mammalian cytoplasm or exosomes (Szabo et al., 2015). CircRNA forms a covalent, closeloop structure lacking both 5′–3′ polarity and a poly-A tail (Lasda and Parker, 2014). This special structure makes circRNAs resistant to degradation by RNA exonucleases, RNase R, and debranching enzymes, more stable than linear transcripts, and less prone to degradation (Suzuki and Tsukahara, 2014; Vicens and Westhof, 2014). The first circRNAs to be identified, viroid, was found in RNA viruses in 1976 (Sanger et al., 1976) and was then found in eukaryotes by electron microscopy in 1979 (Hsu and Coca-Prados, 1979). Previous studies have focused on the biological functions of circRNAs, including their roles as “sponges” for microRNAs via competing endogenous RNA (ceRNA) networks, the circRNA-miRNA-mRNA axis, alternative mRNA splicing patterns via RNA-binding proteins, interactions with RNA polymerase II to regulate transcription, and translation by ribosomes and encoding proteins (Abe et al., 2013; Hansen et al., 2013; Zhang et al., 2013; Du et al., 2016). Recent studies have shown that circRNAs play important regulatory roles in embryonic development, apoptosis, differentiation, and proliferation (Lukiw, 2013; Bachmayr-Heyda et al., 2015; Maiese, 2016). Numerous differentially expressed circRNAs are involved in the development of gastrointestinal diseases and are potential diagnostic and prognostic biomarkers (Torre et al., 2015; Li et al., 2020). Our current understanding of the exact molecular mechanisms by which circRNAs affect the development of ARMs remains limited.

Ethylenethiourea (ETU) is commonly used to create animal models of ARM in rat embryos; typical phenotypes include an abnormally large distance between the urorectal septum and the cloacal membrane, postponed tailgut regression, abnormal apoptosis in the cloacal wall, and maldevelopment of the dorsal cloaca and membrane (Qi et al., 2002; Macedo et al., 2007). Our research group has conducted a series of studies of miRNA and mRNA expression profiles in the development of hindgut in rat embryos (Tang et al., 2014; Long et al., 2018, 2020). However, very little is known about circRNAs in ETU-induced ARMs in rats, and sequence data for circRNA in ETU-induced ARMs have never been systematically described. In the present study, circRNA expression patterns in the hindguts of rat embryos with ETU-induced ARMs were profiled from gestational day (GD)14 to GD16, the key time period for anorectal development (Qi et al., 2002; Bai et al., 2004; Macedo et al., 2007).

To determine the molecular mechanism by which circRNAs operate in ARMs, high-throughput sequencing was used to detect differentially expressed circRNAs in embryonic hindgut tissues of rats with ETU-induced ARMs and normal hindgut tissues. Numerous previously unreported circRNAs were identified, which may improve our understanding of the roles of circRNAs in ARM development. We also constructed ceRNA networks based on target predictions. The aberrant expression of circRNAs detected in the present study improves our understanding of the mechanisms underlying ARMs and provides a valuable basis for future studies of this condition. Furthermore, our data will serve as a useful resource for the development of therapeutic targets and novel diagnostics in the future.

## Materials and Methods

### Ethics Statement

The present study was approved by the Ethics Committee of the China Medical University, Shenyang, China (no. 2015PS213K), and all procedures involving animals were performed in accordance with the guidelines for the care and use of laboratory animals.

### Animals Used and Tissue Collection

Mature female Wistar rats (number: 18; age: 7–8 weeks old; body weight: 230–280 g) were acquired from the Experimental Animal Center, Shengjing Hospital of China Medical University (Shenyang, China). The Key Laboratory of the Health Ministry for Congenital Malformations (Shenyang, China) provided a specific pathogen-free animal laboratory (room temperature: 22 ± 2°C; humidity: 55 ± 5%; 12 h light/12 h dark cycle; free access to water and food) for rodents. The ARM model and hindgut isolation procedure were constructed as per our previous protocol (Tang et al., 2014). In total, 9 pregnant rats were gavage-fed a single dose of 125 mg/kg 1% ETU (Sigma-Aldrich; Merck Millipore, Darmstadt, Germany) on GD10, and the remaining pregnant rats received the same dose of saline without ETU as a control. Cesarean sections were performed on GD14-GD16 to obtain embryos, and the presence of ARMs was determined by light microscopy. The specimens were stored in liquid nitrogen until use. All surgeries were performed after the intraperitoneal injection of sodium pentobarbital for anesthesia. The control group comprised of 252 saline-treated embryos without malformations. All 230 embryos with ETU-induced ARMs had short tails or no tails, and 15 died *in utero*. ARMs were detected in 86.1% (198/230) of ETU-treated embryos.

### RNA Extraction and RNA-Seq

Total RNA was extracted from the specimens using TRIzol (Life Technologies, Carlsbad, CA, United States) according to the manufacturer’s instructions. The RNA was qualified and quantified using an Agilent 2100 Bioanalyzer (Agilent Technologies, Santa Clara, CA, United States). After rRNAs were removed, the rest of RNAs were fragmented using a fragmentation buffer and reverse-transcribed into cDNA with random primers. Second-strand cDNA was synthesized using DNA polymerase I, RNase H, dNTP (dUTP instead of dTTP), and buffer. Next, the cDNA fragments were purified using a QIAquick PCR Extraction Kit (Qiagen, Hilden, Germany), end-repaired, polyadenylated, and ligated to Illumina sequencing adapters. Uracil-*N*-glycosylase was used to digest the second-strand cDNA. After PCR amplification, the digested products were purified using AMPure XP Beads and a High Sensitivity DNA Assay Kit (Agilent Technologies, United States) was used for library quality inspection. RNA sequencing was carried out by Gene Denovo Biotechnology Company (Guangzhou, China) using a HiSeq 2500 system (Illumina, San Diego, CA, United States).

### RNA-Seq Analysis and Identification of circRNAs

To obtain high-quality clean reads, raw reads were filtered as follows: (1) reads containing adapters were removed, (2) reads containing more than 10% unknown nucleotides (N) were removed, and (3) low-quality reads containing more than 50% low-quality bases (*Q*-value ≤ 20) were removed. The efficiency of experimental ribosome RNA removal is affected by the species and sample quality. Therefore, the Bowtie2 short read alignment tool (Langmead and Salzberg, 2012) was used to map the reads to the ribosomal RNA (rRNA) database. The rRNA-mapped reads were removed, followed by mapping to the reference genome using TopHat2 (version 2.1.1) (Kim et al., 2013). After alignment with the reference genome, the reads that could be mapped to the genome were discarded, and the unmapped reads were collected for circRNA identification. The 20-mers from both ends of the unmapped reads were extracted and aligned to the reference genome to identify unique anchor positions within the splice site. Anchored reads that aligned in the reversed orientation (head to tail) indicated circRNA splicing and were subsequently subjected to find_circ (Memczak et al., 2013) to identify circRNAs. The circRNAs were blast searched against circBase (Glažar et al., 2014) for annotation. Those that could not be annotated were considered novel circRNAs. To quantify the circRNAs, back-spliced junction reads were scaled to reads per million mapped reads (RPM) according to the following formula: RPM = 10^6^C/N, where C is the number of back-spliced junction reads that uniquely aligned to a circRNA and N is the total number of back-spliced junction reads.

### Clustering Analysis and Principal Component Analysis (PCA)

The relationships between samples were explored using Unsupervised hierarchical clustering and PCA. Unsupervised hierarchical clustering analyses were performed using gmodels in R^1^ and PCA was conducted using ggplot2 in R^2^.

### Analysis of Differentially Expressed circRNAs

The edgeR software package^3^ was used to identify differentially expressed circRNAs between samples or groups. CircRNAs with an absolute value of fold change of ≥ 2 and a *p*-value of < 0.05 were identified as differentially expressed in comparisons between samples or groups.

### Trend Analysis

To examine patterns of differentially expressed circRNAs, the expression data for each sample were normalized to 0, log2 (v1/v0), and log2 (v2/v0) and clustered using Short Time-series Expression Miner (Ernst and Bar-Joseph, 2006). Clusters with *p* ≤ 0.05 were considered significant.

### Analysis of circRNA-miRNA-mRNA Interactions

**FIGURE 1:**
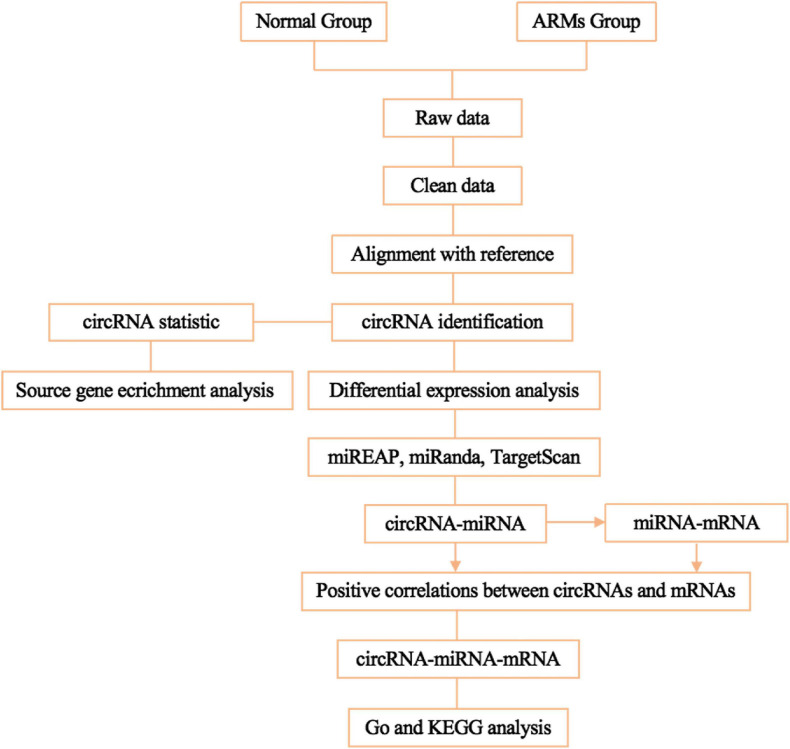
Flowchart of ceRNA network analysis.

To predict mRNAs that interact with circRNAs and miRNAs, miREAP, miRanda, and TargetScan were used, and the intersection between the three prediction algorithms was used as the prediction results for the miRNA target genes. Pairs with positively correlated expression were identified as ceRNA pairs. Negative correlations indicated miRNA-mRNA and miRNA-circRNA pairs. Competition between circRNAs and mRNAs for the same miRNA and positive correlations between circRNAs and mRNAs were then screened. circRNA-miRNA-mRNA networks conforming to the ceRNA rules were visualized using Cytoscape v3.7.1. The flowchart of the circRNA-miRNA-mRNA ceRNA network analysis shown in Figure 1.

### Gene Ontology (GO) and Kyoto Encyclopedia of Genes and Genomes (KEGG) Pathway Analyses

Source genes were mapped to GO terms in the Gene Ontology database^4^, gene numbers were calculated for every term, and significantly enriched GO terms for source genes compared to the genome background were defined using a hypergeometric test. Calculated *p*-values were subjected to false discovery rate (FDR) correction, taking FDR ≤ 0.05 as a threshold for significant enrichment.

KEGG is a major publicly available pathway-related database (Kanehisa et al., 2008). Pathway enrichment analyses were performed to identify significantly enriched metabolic pathways or signal transduction pathways in source genes compared to the whole genome background. The calculated *p*-values were subjected to FDR correction, taking FDR ≤ 0.05 as a threshold for significance. Bubble charts were generated using gglpot2 in R.

### Quantitative Reverse-Transcription Polymerase Chain Reaction (qRT-PCR)

Reverse transcription was performed using a PrimeScript RT Reagent Kit (Takara, Kusatsu, Japan). Fluorescence qRT-PCR was performed using an ABI 7500 detection system (Thermo Fisher Scientific, Waltham, MA, United States). Each 20 μL reaction mixture contained 2 μL of template cDNA, gene-specific primers (0.4 μM), 10 μL of SYBR Premix Ex Taq II (Tli RNaseH Plus, 2×), 0.4 μL of ROX Reference Dye II (50×), and 6 μL of sterilized RNase-free water. The reaction conditions were as follows: 50°C for 2 min, 95°C for 2 min, followed by 40 cycles of 95°C for 15 s and 60°C for 60 s. The relative expression levels of each circRNA normalized against the β-actin level were evaluated using the 2^–ΔΔ*Ct*^ method (Livak and Schmittgen, 2001). qRT-PCR was performed with five biological replicates. Divergent primers were designed to confirm the back-splice junction and were synthesized by Shanghai Sangon Biotech (Supplementary Table 1). The qRT-PCR products were tested by electrophoresis on 2% agarose gels. The amplification products were inserted into a pUCm-T vector for Sanger cloning and sequencing to determine their full length.

### Statistical Analysis

Statistical analyses were performed using SPSS 21.0 (IBM SPSS, Armonk, NY, United States). Statistically significant differences were identified using Student’s *t*-tests (two-tailed). All values are presented as means ± standard deviation. Values of *p* < 0.05 were considered statistically significant.

## Results

### Overview of circRNA-Seq

**FIGURE 2:**
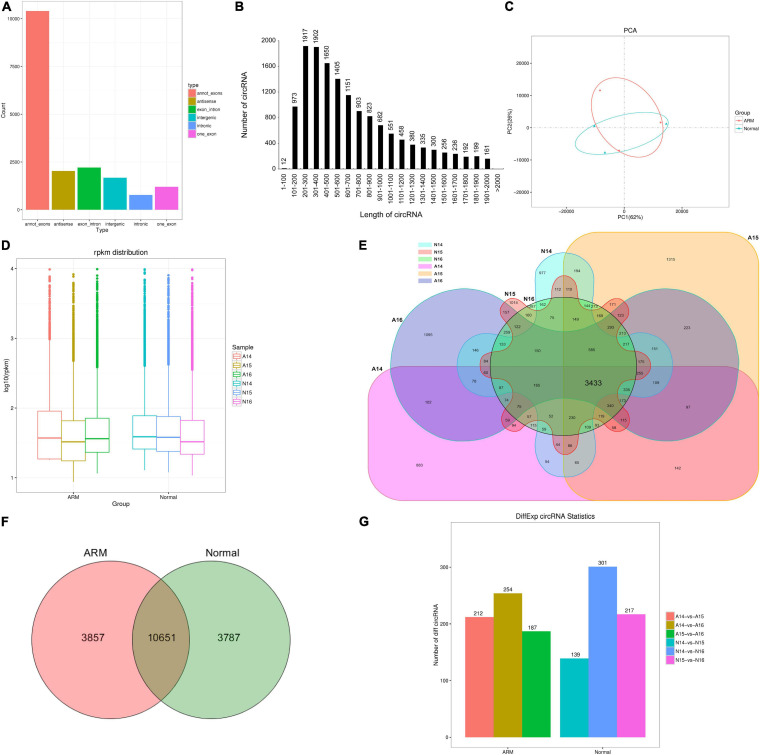
Characterization of circRNAs in rat embryonic hindgut tissues. **(A)** Length distribution of circRNAs. **(B)** Type distribution of circRNAs. **(C)** Principal component analysis (PCA) of different samples at three time points in rat embryonic hindgut tissues from the ARM group and normal group based on the normalized circRNA expression levels. **(D)** Box plots displaying the expression features of circRNAs based on log-transformed expression levels (base 2) in the two groups from GD14–16. **(E,F)** Venn diagram illustrating the overlapping circRNAs detected in the ARM group and normal group. **(G)** Bar graph representing the number of differentially expressed circRNAs in different groups. A, ARM group; N, normal group.

In total, 1,043,148,336 and 1,032,539,926 clean reads were generated in the ARM and control groups, respectively. After removing the low-quality, poly-N-containing, and adapter-containing reads, 915,328,052 and 1,024,161,230 high-quality clean reads were obtained from the ARM and control groups, respectively. After using *find_circ* for mapping, 18,295 circRNAs were obtained. All identified circRNAs were novel. Six types of circRNAs were identified. From most to least frequent, they were categorized as follows: annot_exon, exon_intronmost, antisense, intergenic, one-exon, and intronic (Figure 2A). Most of the circRNAs were approximately 200–400 nucleotides (nt) in length (Figure 2B).

### Time Course Analysis of circRNA Expression Profiles in Hindguts of the ARM and Control Group Rats

An unsupervised hierarchical clustering and PCA were performed to determine the relationships between circRNA expression and spatiotemporal dynamics in the hindguts of normal rat embryos and those with ARMs. The circRNA expression patterns in the two groups formed distinct clusters (Figure 2C). As shown in Figure 2D, there were no significant changes in total circRNA expression levels in the groups over time. The majority of circRNAs annotated in the ARM group were also detected in the normal group (Figure 2E). In total, 10651 circRNAs were co-expressed in both groups, 3857 were expressed exclusively in the ARM group, and 3787 were expressed exclusively in the normal group (Figure 2F).

### circRNAs With Upregulated Expression in the Hindgut During Development

We performed expression profiling to determine whether circRNAs were differentially expressed between the hindguts of the ARM and normal group rats during development. Differences in circRNA expression between groups were calculated based on the back-spliced RPM method. Only differentially expressed circRNAs with an absolute value of fold change of ≥ 2 and a *p*-value of < 0.05 were considered.

**FIGURE 3:**
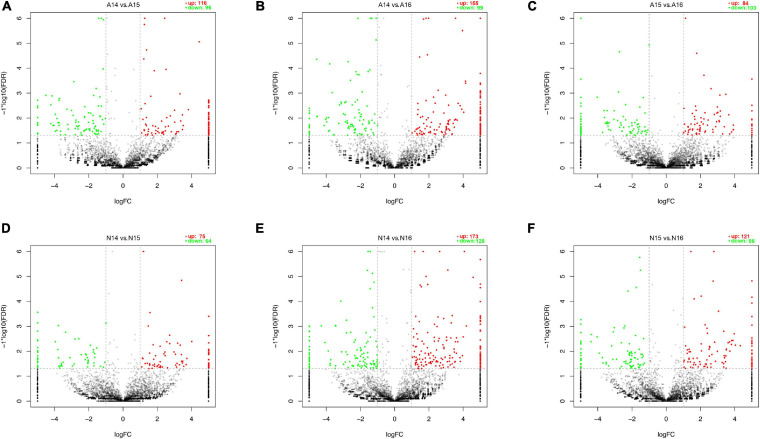
Volcano plots showing up- and downregulated circRNAs in the ARM group and normal group. **(A–F)** Red dots represent upregulated and green dots represent downregulated circRNAs. A, ARM group; N, normal group.

We performed pairwise comparisons of circRNAs between stages in the ARM group and normal group. The number of differentially expressed circRNAs ranged from 139 to 301 during development in the two groups (Figure 2G). Volcano plots indicated that the circRNAs tended to be upregulated in the normal group during development, but only tended to be upregulated at GD14 vs. GD15 and GD14 vs. GD16 in the ARM group. In particular, in the ARM group, 96 circRNAs (0.9%) were significantly downregulated and 116 (1.1%) were significantly upregulated at GD14 vs. GD15 (Figure 3A), 99 (0.9%) were significantly downregulated and 155 (1.5%) were significantly upregulated in GD14 vs. GD16 (Figure 3B), and 103 (1.0%) were significantly downregulated and 84 (0.8%) were significantly upregulated in GD15 vs. GD16 (Figure 3C). In the normal group, 64 circRNAs (0.6%) were significantly downregulated and 75 (0.7%) were significantly upregulated in GD14 vs. GD15 (Figure 3D), 128 (1.2%) were significantly downregulated and 173 (1.6%) were significantly upregulated in GD14 vs. GD16 (Figure 3E), and 96 (0.9%) were significantly downregulated and 121 (1.1%) were significantly upregulated in GD15 vs. GD16 (Figure 3F). These results indicated that there is spatiotemporal variation in the expression levels of circRNAs.

### Dynamics of circRNA Expression in the Hindguts of Rats in the ARM and Normal Groups Over Time

**FIGURE 4:**
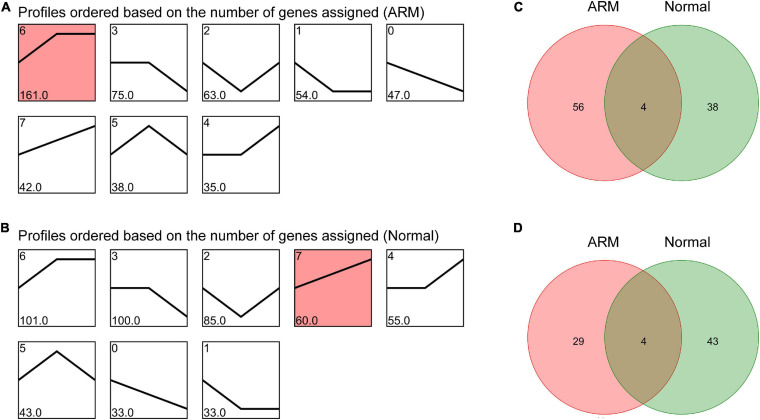
Overall expression patterns of circRNAs in the ARM group and normal group. **(A)** Expression patterns of circRNAs in the ARM group. **(B)** Expression patterns of circRNAs in the normal group. Red indicates significant enrichment (*p* < 0.05). The profile IDs are shown in the upper left corner, and the number of genes in the profile is shown in the lower left corner. **(C)** Venn diagram showing upregulated circRNAs in both the ARM group and normal group. **(D)** Venn diagram showing downregulated circRNAs in both the ARM group and normal group.

To characterize the changes in circRNA expression, we evaluated trends in the highly differentially expressed circRNAs in each group during development. We identified seven main circRNA profiles in the ARM and normal groups, and each showed a characteristic expression pattern (referred to as Profiles 1–7). Profile 6 was significant in the ARM group (Figure 4A) and Profile 7 was significantly upregulated in the normal group (Figure 4B). We also analyzed the up- and downregulated circRNAs in both groups. We found that the expression levels of 60 circRNAs were higher in the normal group and 42 were higher in the ARM group (Figure 4C), whereas levels of 33 circRNAs were lower in the normal group and 47 were lower in the ARM group (Figure 4D). These findings suggested that the dysregulation of circRNAs plays a role in hindgut development.

### Screening of Differentially Expressed circRNAs and Clustering Analysis

**FIGURE 5:**
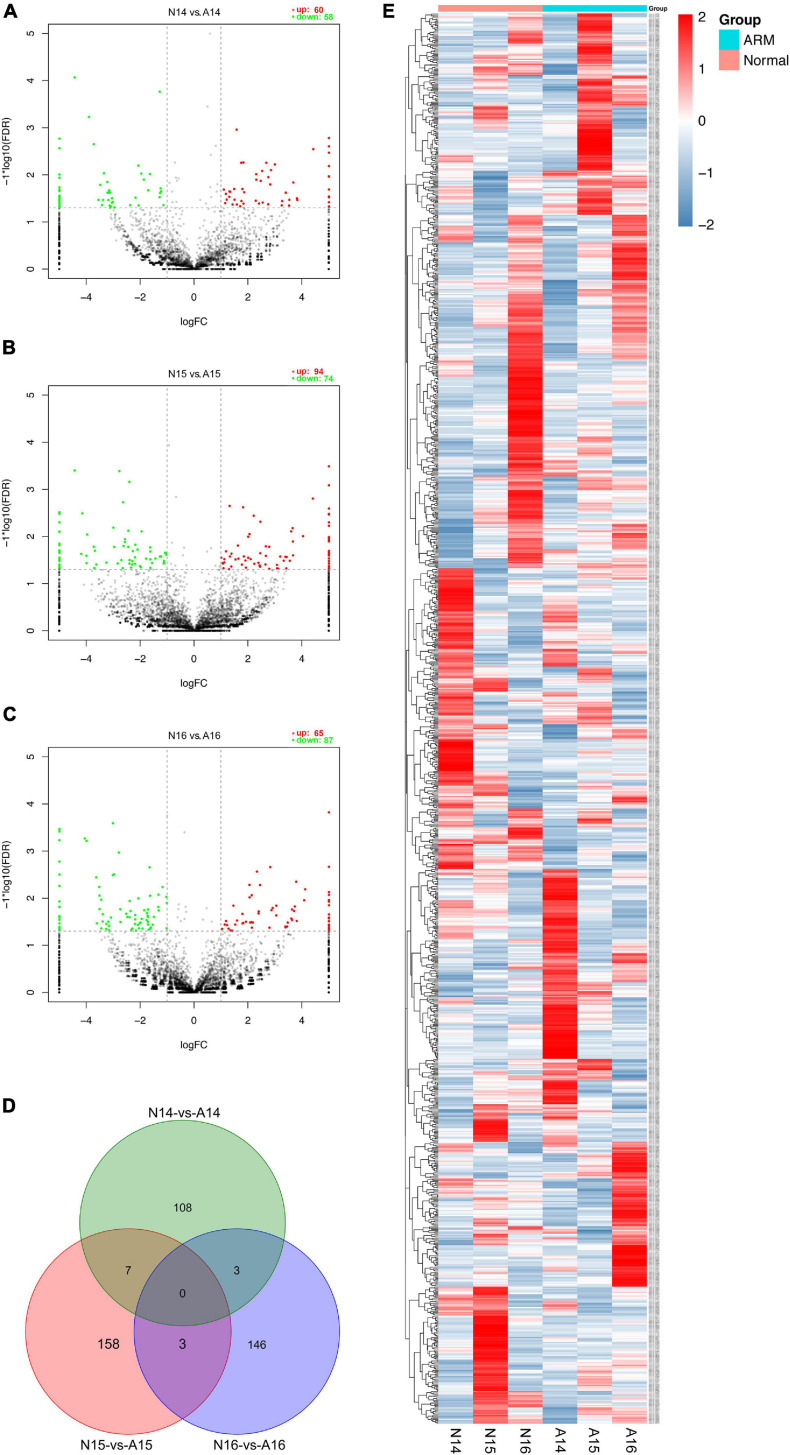
**(A–C)** Volcano plots showing the up- and downregulated circRNAs in the ARM group and normal group. Red dots represent upregulated and green dots represent downregulated circRNAs. **(D)** Venn diagram showing the overlap among these circRNAs between the three time-points. **(E)** Hierarchical cluster analysis and heat map showing differentially expressed circRNAs between the ARM group and normal group. Colors from blue to red indicate circRNA expression levels from low to high. Each column in the heat map represents a mean of three biological replicates, and each row represents a circRNA. A, ARM group; N, normal group.

The expression profiles in hindgut samples from the normal and ARM groups were compared at key time-points during anus formation (GD14, GD15, and GD16). There were significant differences in the expression levels of 425 circRNAs (|Fc| > 2, *p* < 0.05). Compared to the normal group, 60 circRNAs were significantly upregulated and 58 were significantly downregulated in the ARM group on GD14, 94 circRNAs were significantly upregulated and 74 were significantly downregulated in the ARM group on GD15, and 65 circRNAs were significantly upregulated and 87 were significantly downregulated in the ARM group on GD16 (Figures 5A–C). The overlap among these circRNAs between the three time-points is shown in Figure 5D. A heat map of significant differential expression is shown in Figure 5E.

### Predicted ceRNA Networks

**FIGURE 6:**
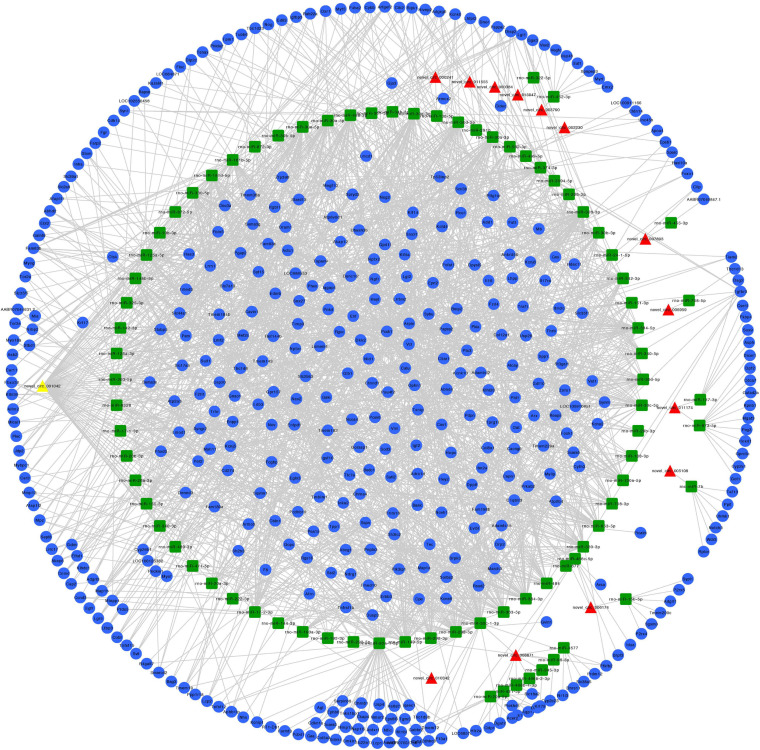
Predicted circRNA-miRNA-mRNA networks of circRNAs with increased expression in rat embryonic hindgut tissues in the ARM group compared with the normal group. Triangular red nodes represent up-regulated circRNAs, square green nodes represent miRNAs, and circular blue nodes represent mRNAs. Straight lines indicate interactions between miRNA and circRNA or mRNAs.

**FIGURE 7:**
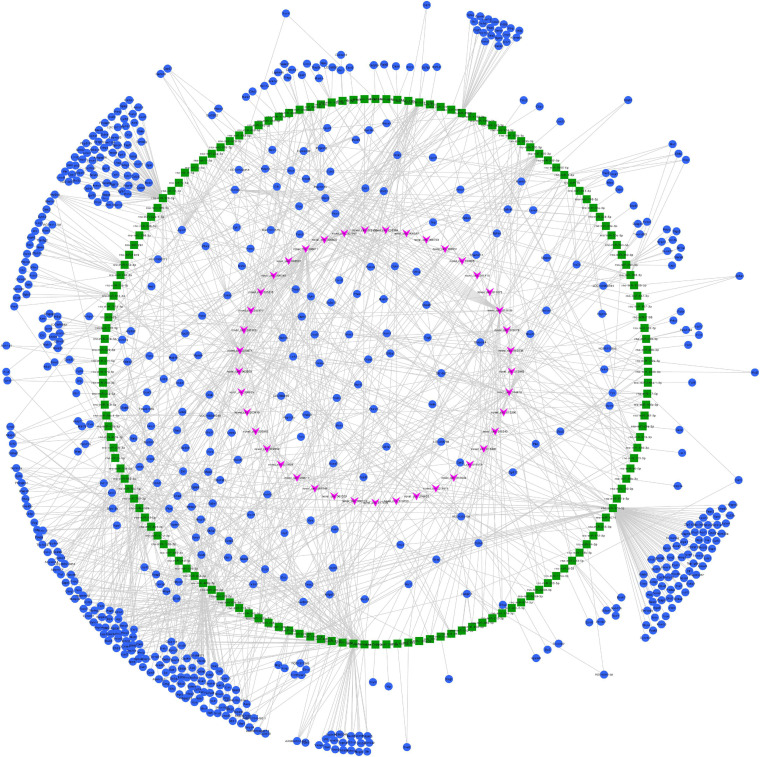
Predicted circRNA-miRNA-mRNA networks of circRNAs with decreased expression in rat embryonic hindgut tissues in the ARM group compared with the normal group. Triangular pink nodes represent down-regulated circRNAs, square green nodes represent miRNAs, and circular blue nodes represent mRNAs. Straight lines indicate interactions between miRNA and circRNA or mRNAs.

We constructed ceRNA networks based on 425 differentially expressed circRNAs. According to StarBase v3.0 and TargetScan v7.2, among differentially expressed circRNAs, 55 (14 up- and 41 downregulated) were predicted to bind to 195 miRNAs and 947 mRNAs. The detailed information of the 55 differentially expressed circRNAs at the three time-points are shown in Table 1. The circRNA-miRNA-mRNA correlations were visualized using Cytoscape 3.7.1 (Figures 6, 7). According to a connectivity analysis, the top five circRNAs were novel_circ_001042, novel_circ_012639, novel_circ_003974, novel_circ_006611, and novel_circ_014878. novel_circ_001042 was predicted to interact with 64 miRNAs and 328 mRNAs. These included the wingless-type MMTV integration site family (WNT) signaling pathway-related genes *Fzd4*, *Ccnd3*, and *Dkk2*, the transforming growth factor-β (TGF-β) signaling pathway-related gene *Bmp2*, and the mitogen-activated protein kinase (MAPK) signaling pathway-related genes *Flnc*, *Egfr*, *Mef2c*, *Dusp3*, and *Cacng6*. This complicated ceRNA network suggested that novel_circ_001042 played regulatory roles in various pathways via miRNAs and their targets during hindgut development.

**TABLE 1:** Differentially expressed circRNAs.

ID	log2 (fold change)	*P*-value
**GD14; N vs. A**		
novel_circ_005120	–16.80445315	0.035641889
novel_circ_006591	–3.120244448	0.023688892
novel_circ_003974	–3.053221315	0.039032126
novel_circ_011675	–2.176357531	0.030632664
novel_circ_006809	–1.645158719	0.009663424
novel_circ_002034	–1.285355276	0.029852901
novel_circ_008138	–1.278446316	0.000172223
novel_circ_002017	–1.265940918	0.024316916
novel_circ_003869	–1.224397979	0.022398961
novel_circ_011174	1.853502957	0.023384327
novel_circ_007893	2.299616362	0.009735364
novel_circ_002230	3.245529718	0.044635454
novel_circ_003790	16.73277898	0.046924131
novel_circ_005108	16.74691874	0.047852203
novel_circ_010342	16.97284673	0.029985969
**GD15; N vs. A**		
novel_circ_000115	–16.5911656	0.047741235
novel_circ_011115	–17.17468259	0.045157085
novel_circ_009205	–16.9621121	0.031863695
novel_circ_002552	–16.71470825	0.029834198
novel_circ_017476	–16.61250487	0.022203997
novel_circ_012938	–2.837516461	0.037442792
novel_circ_001300	–2.775952353	0.000408425
novel_circ_007019	–2.651840519	0.04731267
novel_circ_016600	–2.569746484	0.011431925
novel_circ_015949	–2.441524228	0.016680423
novel_circ_008708	–2.440559967	0.007597276
novel_circ_012639	–2.404748731	0.000694139
novel_circ_014878	–2.277915194	0.020484521
novel_circ_003419	–1.631402216	0.020883386
novel_circ_016730	–1.273701762	0.026751436
novel_circ_003835	–1.116177089	0.036829487
novel_circ_011174	3.546260852	0.032843917
novel_circ_008671	16.28869484	0.049117697
novel_circ_000241	16.59350112	0.02397067
novel_circ_015947	16.62866004	0.025156143
novel_circ_000384	16.90450162	0.024117077
novel_circ_006959	17.35124018	0.000821534
**GD16; N vs. A**		
novel_circ_005340	–17.52540703	0.000587897
novel_circ_015300	–17.15518294	0.003161602
novel_circ_015242	–16.60247339	0.015429026
novel_circ_009151	–16.42630727	0.028601564
novel_circ_002915	–16.23693974	0.045883863
novel_circ_001648	–3.636266866	0.003621806
novel_circ_015200	–3.609065544	0.034131194
novel_circ_008532	–2.732468102	0.032040587
novel_circ_005215	–2.15723716	0.048959819
novel_circ_008364	–2.047701005	0.043846608
novel_circ_006611	–1.854643226	0.018081202
novel_circ_012678	–1.713763152	0.025258653
novel_circ_015519	–1.626743877	0.04439456
novel_circ_016175	−1.174967821	0.005791023
novel_circ_005123	−1.03502976	0.009447818
novel_circ_013949	−1.018853757	0.043790165
novel_circ_001042	1.180791996	0.030647319
novel_circ_006174	2.173073874	0.032982196
novel_circ_011555	16.57119281	0.048915297

*GD, gestational day; N, normal group; A, anorectal malformatios group.*

### Enrichment of Differentially Expressed circRNAs

**FIGURE 8:**
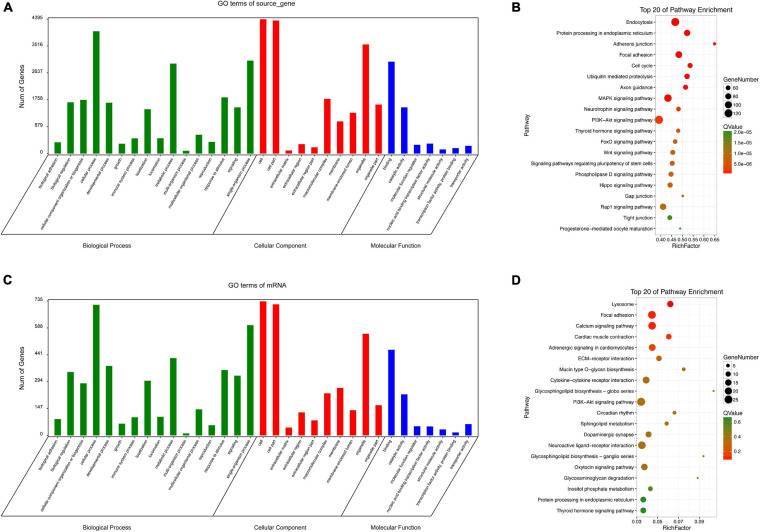
GO and KEGG analyses. GO categories **(A)** and KEGG pathways **(B)** for genes related to differentially expressed circRNAs source genes in rat hindgut tissues from the ARM group compared with the normal group. GO categories **(C)** and KEGG pathways **(D)** for differential circRNA-miRNA target genes in rat embryonic hindgut tissues from the ARM group and the normal group. *Q*-values are normalized *p*-values indicating the significance of the enrichment.

Since circRNAs can directly regulate source genes, we conducted an enrichment analysis of source genes of circRNAs. According to a GO analysis, circRNA source genes were enriched for 33 GO terms in the three main categories, biological processes, molecular functions, and cellular components (Figure 8A). The top 20 KEGG pathways are listed in Figure 8B. The enriched pathways included the WNT pathway and the MAPK pathway, which are closely associated with embryonic development and ARMs.

For non-coding RNAs, functional annotations are determined by the functions of associated mRNAs. We evaluated potential functions of significantly up-regulated and down-regulated circRNAs in the ARM and normal groups by an enrichment analysis of target genes. Based on a GO analysis, the target genes related to the circRNA signature were involved in various biological processes, including single-organism processes, cellular processes, and metabolic process (Figure 8C). In a KEGG pathway analysis, endocytosis and PI3K-Akt were identified as the most significant pathways (Figure 8D).

### Validation of Sequencing Data

To validate the circRNAs identified by RNA-Seq, we randomly selected 7 up- and 7 downregulated circRNAs for validation by qRT-PCR. Among the 14 differentially expressed circRNAs, novel_circ_008138, novel_circ_002017, novel_circ_010342, novel_circ_002202, and novel_circ_016535 were differentially expressed on GD14; novel_circ_012938, novel_circ_01300, novel_circ_016600, and novel_circ_015194 were differentially expressed on GD15; novel_circ_016175, novel_circ_008364, novel_circ_017060, and novel_circ_013064 were differentially expressed on GD16; and novel_circ_011174 was differentially expressed at two time-points (GD14 and GD15) (*p* < 0.05; Supplementary Figure 1A). The relative expression levels were consistent with the RNA-Seq data. Sanger cloning and sequencing results for novel_circ_002017 and novel_circ_008138 are shown in Supplementary Figures 1B,C. Agarose gels electrophoresis was used to validate that a single product had been amplified are shown in Supplementary Figure 1D.

## Discussion

ARMs are complex deformities determined by many factors. Genetic factors have an important influence on the etiology of ARMs (Khanna et al., 2018). The roles of circRNA in embryonic development and congenital malformations of the gastrointestinal system have rarely been reported. Different from most RNAs involved in translation, circRNAs often act at the level of gene expression (Esteller, 2011). The versatility of circRNA expression is mainly reflected in post-transcriptional regulation, which is usually achieved by RNA–RNA interactions (Guil and Esteller, 2015). Studies of circRNAs related to gastrointestinal development are very limited. High-throughput sequencing has confirmed that circRNA expression levels differ among anatomical parts of the digestive tract, and these differences may be closely related to cell proliferation and differentiation during development (Danan et al., 2012; Salzman et al., 2013; Shen et al., 2017). Increasing number of studies have shown that circRNAs, acting as miRNA sponges, inhibit miRNAs and eventually mediate the expression of mRNAs (Hansen et al., 2013). Peng et al. (2017) found that circ-ZNF609 participates in the pathogenesis of congenital megacolon via the mir-150-5P/AKT3 pathway. Zhou et al. performed a sequencing analysis of human congenital megacolon specimens and found that the down-regulation of circ-PRKCI could inhibit cell proliferation and differentiation and inhibit the expression of the mir-1324 target gene *PLCB1* (Zhou et al., 2018). However, the precise circRNAs with key regulatory roles in the normal development of the anus and rectum and the mechanism by which these circRNAs affect the pathogenesis of anorectal atresia and rectourethral fistula are unclear. Therefore, further research is required on this topic.

According to previous morphological studies, urorectal fistulas in rat embryos with ARMs can be explained by a failure of the fusion of the urorectal septum with the cloacal membrane during the critical period of anorectal development. Deduced proliferation and massive apoptosis play important roles in the development of ARMs (Qi et al., 2000a, b, 2002). In the present study, 18,295 circRNAs were identified at key time-points (GD14, GD15, and GD16) during anorectal development in the rat hindgut by high-throughput sequencing. We analyzed expression patterns and performed a bioinformatics analysis of circRNAs that may be involved in the pathogenesis of ARMs. We detected aberrantly expressed circRNAs in a comparison of rat fetuses with ARMs and normal rat fetuses. After screening (|Fc| > 2, *p* < 0.05), we detected 118, 168, and 152 differentially expressed circRNAs at GD14, GD15, and GD16, respectively. We can infer that there is a temporal imbalance in circRNA expression.

Using a bioinformatics approach for target prediction, among 425 differentially expressed circRNAs, 55 had ceRNA network connectivity. Compared to the normal group, 14 genes were upregulated and 41 genes were downregulated, suggesting that reductions in circRNA levels were dominant in the ARM group and might contribute to the development of ARMs. A circRNA-miRNA-mRNA ceRNA network was also constructed. A KEGG pathway enrichment analysis of miRNA-target genes identified the WNT, TGF-β, and MAPK signaling pathways, which are closely related to ARM (Nakamura et al., 2007; Wong et al., 2013; Wang et al., 2015). GO terms of related genes provided compelling evidence that circRNAs can influence metabolic, regulatory, and cellular functions. Our results demonstrated that these differentially expressed circRNAs may play important roles in the development of ETU-induced ARMs.

As a member of the miR-17-92 family, miR-92 plays an important role in the regulation of early embryonic development as well as in tumorigenesis and differentiation in various cancers (Monzo et al., 2008; Jevnaker et al., 2011). Our research group has previously shown that miR-92a-2-5p could have a regulatory role in ARMs. The overexpression of miR-92a-2-5p leads to a decrease in proliferation and an increase in apoptosis in an intestinal cell line (Long et al., 2020). Our results based on RNA-seq data showed that rno-miR-92a-1-5p is related to the regulation of 90 mRNAs. novel_circ_001042, novel_circ_003974, and novel_circ_010342 were associated with rno-miR-92a-1-5p and may function as ceRNAs to regulate the progression of ARMs. Notably, most of the circRNAs identified in this analysis have not been studied previously. Therefore, further research should explore the relationships between circRNAs/miRNAs and ARMs based on our ceRNA-associated network.

## Conclusion

In conclusion, a high-throughput sequencing approach was used to identify numerous differentially expressed circRNAs between the hindguts of normal rat embryos and those with ARMs on GD14–GD16. Using a bioinformatics approach, a circRNA-miRNA-mRNA network was constructed. The potential target genes of novel_circ_001042 may be closely related to the development of ARMs. circRNAs are candidate biomarkers for disease diagnosis and therapeutic targets owing to their high conservation, stability, and tissue-specificity. Our results provide novel insight into the roles of circRNAs in the development of ARMs and provide a valuable resource for further analyses of molecular mechanisms and signaling networks. The construction of regulatory networks could improve our understanding of the basic molecular mechanisms underlying ARM.

## Data Availability Statement

The datasets generated for this study can be found in NCBI GEO accession GSE159306: go to https://www.ncbi.nlm.nih.gov/geo/query/acc.cgi?acc=GSE159306.

## Ethics Statement

The animal study was reviewed and approved by the Ethnical Committee on Science and New Techniques of Shengjing Hospital of China Medical University.

## Author Contributions

All authors listed have made a substantial, direct and intellectual contribution to the work, and approved it for publication.

## Conflict of Interest

The authors declare that the research was conducted in the absence of any commercial or financial relationships that could be construed as a potential conflict of interest.

## Footnotes

1 https://cran.r-project.org/web/packages/gmodels/index.html

2 https://cran.r-project.org/web/packages/ggplot2/index.html

3 http://www.r-project.org/

4 http://www.geneontology.org/

## Supplementary Material

Supplementary Figure 1 **(A)** Fourteen circRNAs in rat embryonic hindgut tissues in the ARM group and normal group were validated by qRT-PCR. ^∗^*p* < 0.05, *n* = 5. **(B,C)** Two representative examples (novel_circ_002017 and novel_circ_008138) of qRT-PCR products confirmed by Sanger cloning and sequencing. **(D)** qRT-PCR products were visualized using agarose gels electrophoresis. GD, gestational day; A, ARM group; N, normal group.

Supplementary Table 1 The sequences of the primers pairs.

## Abbreviations

ARMs: anorectal malformations
ETU: ethylenethiourea
GD: gestational day
Rpm: reads per million mapped reads
circRNAs: circular RNAs
ceRNA: competing endogenous RNA
rRNA: ribosomal RNA
GO: Gene Ontology
KEGG: Kyoto Encyclopedia of Genes and Genomes
FDR: false discovery rate
qRT-PCR: quantitative reverse-transcription polymerase chain reaction
WNT: wingless-type MMTV integration site family
TGF- β: transforming growth factor- β
MAPK: mitogen-activated protein kinase
GEO: Gene Expression Omnibus
PCA: principal component analysis.

## References

[B1] Abe N. Hiroshima M. Maruyama H. Nakashima Y. Nakano Y. Matsuda A. (2013). Rolling circle amplification in a prokaryotic translation system using small circular RNA. *Angew. Chem. Int. Ed. Engl.* 52 7004–7008. 10.1002/anie.201302044 23716491

[B2] Bachmayr-Heyda A. Reiner A. T. Auer K. Sukhbaatar N. Aust S. Bachleitner-Hofmann T. (2015). Correlation of circular RNA abundance with proliferation–exemplified with colorectal and ovarian cancer, idiopathic lung fibrosis, and normal human tissues. *Sci. Rep.* 5:8057. 10.1038/srep08057 25624062 PMC4306919

[B3] Bai Y. Chen H. Yuan Z. W. Wang W. (2004). Normal and abnormal embryonic development of the anorectum in rats. *J. Pediatr. Surg.* 39 587–590. 10.1016/j.jpedsurg.2003.12.002 15065033

[B4] Cuschieri A. Eurocat Working Group. (2001). Descriptive epidemiology of isolated anal anomalies: a survey of 4.6 million births in Europe. *Am. J. Med. Genet.* 103 207–215. 10.1002/ajmg.1532.abs 11745992

[B5] Danan M. Schwartz S. Edelheit S. Sorek R. (2012). Transcriptome-wide discovery of circular RNAs in Archaea. *Nucleic Acids Res.* 40 3131–3142. 10.1093/nar/gkr1009 22140119 PMC3326292

[B6] Draaken M. Prins W. Zeidler C. Hilger A. Mughal S. S. Latus J. (2012). Involvement of the WNT and FGF signaling pathways in non-isolated anorectal malformations: sequencing analysis of WNT3A, WNT5A, WNT11, DACT1, FGF10, FGFR2 and the T gene. *Int. J. Mol. Med.* 30 1459–1464. 10.3892/ijmm.2012.1124 22961180

[B7] Du W. W. Yang W. Liu E. Yang Z. Dhaliwal P. Yang B. B. (2016). Foxo3 circular RNA retards cell cycle progression via forming ternary complexes with p21 and CDK2. *Nucleic Acids Res.* 44 2846–2858. 10.1093/nar/gkw027 26861625 PMC4824104

[B8] Endo M. Hayashi A. Ishihara M. Maie M. Nagasaki A. Nishi T. (1999). Analysis of 1,992 patients with anorectal malformations over the past two decades in Japan. Steering Committee of Japanese Study Group of Anorectal Anomalies. *J. Pediatr. Surg.* 34 435–441. 10.1016/s0022-3468(99)90494-3 10211649

[B9] Ernst J. Bar-Joseph Z. (2006). STEM: a tool for the analysis of short time series gene expression data. *BMC Bioinform.* 7:191. 10.1186/1471-2105-7-191 16597342 PMC1456994

[B10] Esteller M. (2011). Non-coding RNAs in human disease. *Nat. Rev. Genet.* 12 861–874. 10.1038/nrg3074 22094949

[B11] Glažar P. Papavasileiou P. Rajewsky N. (2014). circBase: a database for circular RNAs. *RNA* 20 1666–1670. 10.1261/rna.043687.113 25234927 PMC4201819

[B12] Grano C. Bucci S. Aminoff D. Lucidi F. Violani C. (2013). Quality of life in children and adolescents with anorectal malformation. *Pediatr. Surg. Int.* 29 925–930. 10.1007/s00383-013-3359-8 23907176

[B13] Guil S. Esteller M. (2015). RNA-RNA interactions in gene regulation: the coding and noncoding players. *Trends Biochem. Sci.* 40 248–256. 10.1016/j.tibs.2015.03.001 25818326

[B14] Hansen T. B. Jensen T. I. Clausen B. H. Bramsen J. B. Finsen B. Damgaard C. K. (2013). Natural RNA circles function as efficient microRNA sponges. *Nature* 495 384–388. 10.1038/nature11993 23446346

[B15] Hsu M. T. Coca-Prados M. (1979). Electron microscopic evidence for the circular form of RNA in the cytoplasm of eukaryotic cells. *Nature* 280 339–340. 10.1038/280339a0 460409

[B16] Jevnaker A. M. Khuu C. Kjøle E. Bryne M. Osmundsen H. (2011). Expression of members of the miRNA17-92 cluster during development and in carcinogenesis. *J. Cell. Physiol.* 226 2257–2266. 10.1002/jcp.22562 21660949

[B17] Kanehisa M. Araki M. Goto S. Hattori M. Hirakawa M. Itoh M. (2008). KEGG for linking genomes to life and the environment. *Nucleic Acids Res.* 36 D480–D484. 10.1093/nar/gkm882 18077471 PMC2238879

[B18] Khanna K. Sharma S. Pabalan N. Singh N. Gupta D. K. (2018). A review of genetic factors contributing to the etiopathogenesis of anorectal malformations. *Pediatr. Surg. Int.* 34 9–20. 10.1007/s00383-017-4204-2 29094201

[B19] Kim D. Pertea G. Trapnell C. Pimentel H. Kelley R. Salzberg S. L. (2013). TopHat2: accurate alignment of transcriptomes in the presence of insertions, deletions and gene fusions. *Genome Biol.* 14:R36. 10.1186/gb-2013-14-4-r36 23618408 PMC4053844

[B20] Langmead B. Salzberg S. L. (2012). Fast gapped-read alignment with Bowtie 2. *Nat. Methods* 9 357–359. 10.1038/nmeth.1923 22388286 PMC3322381

[B21] Lasda E. Parker R. (2014). Circular RNAs: diversity of form and function. *RNA* 20 1829–1842. 10.1261/rna.047126.114 25404635 PMC4238349

[B22] Li R. Jiang J. Shi H. Qian H. Zhang X. Xu W. (2020). CircRNA: a rising star in gastric cancer. *Cell. Mol. Life Sci.* 77 1661–1680. 10.1007/s00018-019-03345-5 31659415 PMC11104848

[B23] Livak K. J. Schmittgen T. D. (2001). Analysis of relative gene expression data using real-time quantitative PCR and the 2(-Delta Delta C(T)) Method. *Methods* 25 402–408. 10.1006/meth.2001.1262 11846609

[B24] Long C. Y. Tang X. B. Wang W. L. Yuan Z. W. Bai Y. Z. (2018). Microarray analysis of miRNAs during hindgut development in rat embryos with ethylenethiourea-induced anorectal malformations. *Int. J. Mol. Med.* 42 2363–2372. 10.3892/ijmm.2018.3809 30106085 PMC6192757

[B25] Long C. Y. Xiao Y. X. Li S. Y. Tang X. B. Yuan Z. W. Bai Y. Z. (2020). Upregulation of miR-92a-2-5p potentially contribute to anorectal malformations by inhibiting proliferation and enhancing apoptosis via PRKCA/β-catenin. *Biomed. Pharmacother.* 127:110117. 10.1016/j.biopha.2020.110117 32244197

[B26] Lukiw W. J. (2013). Circular RNA (circRNA) in Alzheimer’s disease (AD). *Front. Genet.* 4:307. 10.3389/fgene.2013.00307 24427167 PMC3875874

[B27] Macedo M. Martins J. L. Meyer K. F. (2007). Evaluation of an experimental model for anorectal anomalies induced by ethylenethiourea. *Acta Cir. Bras.* 22 130–136. 10.1590/s0102-86502007000200010 17375220

[B28] Maiese K. (2016). Disease onset and aging in the world of circular RNAs. *J. Trans. Sci.* 2 327–329. 10.15761/jts.1000158 27642518 PMC5026119

[B29] Memczak S. Jens M. Elefsinioti A. Torti F. Krueger J. Rybak A. (2013). Circular RNAs are a large class of animal RNAs with regulatory potency. *Nature* 495 333–338. 10.1038/nature11928 23446348

[B30] Monzo M. Navarro A. Bandres E. Artells R. Moreno I. Gel B. (2008). Overlapping expression of microRNAs in human embryonic colon and colorectal cancer. *Cell Res.* 18 823–833. 10.1038/cr.2008.81 18607389

[B31] Nah S. A. Ong C. C. Lakshmi N. K. Yap T. L. Jacobsen A. S. Low Y. (2012). Anomalies associated with anorectal malformations according to the Krickenbeck anatomic classification. *J. Pediatr. Surg.* 47 2273–2278. 10.1016/j.jpedsurg.2012.09.017 23217888

[B32] Nakamura T. Tsuchiya K. Watanabe M. (2007). Crosstalk between Wnt and Notch signaling in intestinal epithelial cell fate decision. *J. Gastroenterol.* 42 705–710. 10.1007/s00535-007-2087-z 17876539

[B33] Peng L. Chen G. Zhu Z. Shen Z. Du C. Zang R. (2017). Circular RNA ZNF609 functions as a competitive endogenous RNA to regulate AKT3 expression by sponging miR-150-5p in Hirschsprung’s disease. *Oncotarget* 8 808–818. 10.18632/oncotarget.13656 27903978 PMC5352198

[B34] Qi B. Q. Beasley S. W. Frizelle F. A. (2002). Clarification of the processes that lead to anorectal malformations in the ETU-induced rat model of imperforate anus. *J. Pediatr. Surg.* 37 1305–1312. 10.1053/jpsu.2002.34996 12194121

[B35] Qi B. Q. Beasley S. W. Williams A. K. Fizelle F. (2000a). Apoptosis during regression of the tailgut and septation of the cloaca. *J. Pediatr. Surg.* 35 1556–1561. 10.1053/jpsu.2000.18309 11083422

[B36] Qi B. Q. Williams A. Beasley S. Frizelle F. (2000b). Clarification of the process of separation of the cloaca into rectum and urogenital sinus in the rat embryo. *J. Pediatr. Surg.* 35 1810–1816. 10.1053/jpsu.2000.19265 11101742

[B37] Rintala R. J. (2016). Congenital cloaca: long-term follow-up results with emphasis on outcomes beyond childhood. *Semin. Pediatr. Surg.* 25 112–116. 10.1053/j.sempedsurg.2015.11.011 26969236

[B38] Salzman J. Chen R. E. Olsen M. N. Wang P. L. Brown P. O. (2013). Cell-type specific features of circular RNA expression. *PLoS Genet.* 9:e1003777. 10.1371/journal.pgen.1003777 24039610 PMC3764148

[B39] Sanger H. L. Klotz G. Riesner D. Gross H. J. Kleinschmidt A. K. (1976). Viroids are single-stranded covalently closed circular RNA molecules existing as highly base-paired rod-like structures. *Proc. Natl. Acad. Sci. U.S.A.* 73 3852–3856. 10.1073/pnas.73.11.3852 1069269 PMC431239

[B40] Shen Y. Guo X. Wang W. (2017). Identification and characterization of circular RNAs in zebrafish. *FEBS Lett.* 591 213–220. 10.1002/1873-3468.12500 27878987

[B41] Stoll C. Alembik Y. Dott B. Roth M. P. (2007). Associated malformations in patients with anorectal anomalies. *Eur. J. Med. Genet.* 50 281–290. 10.1016/j.ejmg.2007.04.002 17572165

[B42] Suzuki H. Tsukahara T. (2014). A view of pre-mRNA splicing from RNase R resistant RNAs. *Int. J. Mol. Sci.* 15 9331–9342. 10.3390/ijms15069331 24865493 PMC4100097

[B43] Szabo L. Morey R. Palpant N. J. Wang P. L. Afari N. Jiang C. (2015). Statistically based splicing detection reveals neural enrichment and tissue-specific induction of circular RNA during human fetal development. *Genome Biol.* 16:126. 10.1186/s13059-015-0690-5 26076956 PMC4506483

[B44] Tang X. B. Zhang T. Wang W. L. Yuan Z. W. Bai Y. Z. (2014). Temporal and spatial expression of caudal-type homeobox gene-2 during hindgut development in rat embryos with ethylenethiourea-induced anorectal malformations. *Cells Tissues Res.* 357 83–90. 10.1007/s00441-014-1858-0 24744267

[B45] Torre L. A. Bray F. Siegel R. L. Ferlay J. Lortet-Tieulent J. Jemal A. (2015). Global cancer statistics, 2012. *CA Cancer J. Clin.* 65 87–108. 10.3322/caac.21262 25651787

[B46] Vicens Q. Westhof E. (2014). Biogenesis of circular RNAs. *Cell* 159 13–14. 10.1016/j.cell.2014.09.005 25259915

[B47] Wang C. Li L. Cheng W. (2015). Anorectal malformation: the etiological factors. *Pediatr. Surg. Int.* 31 795–804. 10.1007/s00383-015-3685-0 25899933

[B48] Wijers C. H. van Rooij I. A. Marcelis C. L. Brunner H. G. de Blaauw I. Roeleveld N. (2014). Genetic and nongenetic etiology of nonsyndromic anorectal malformations: a systematic review. *Birth Defects Res. C Embryo Today* 102 382–400. 10.1002/bdrc.21068 25546370

[B49] Wong E. H. Ng C. L. Lui V. C. So M. T. Cherny S. S. Sham P. C. (2013). Gene network analysis of candidate loci for human anorectal malformations. *PLoS One* 8:e69142. 10.1371/journal.pone.0069142 23936318 PMC3731316

[B50] Wood R. J. Levitt M. A. (2018). Anorectal Malformations. *Clin. Colon. Rectal. Surg.* 31 61–70. 10.1055/s-0037-1609020 29487488 PMC5825858

[B51] Zhang Y. Zhang X. O. Chen T. Xiang J. F. Yin Q. F. Xing Y. H. (2013). Circular intronic long noncoding RNAs. *Mol. Cell* 51 792–806. 10.1016/j.molcel.2013.08.017 24035497

[B52] Zhou L. Li Y. Jiang W. Zhang H. Wen Z. Su Y. (2018). Down-regulation of circ-PRKCI inhibits cell migration and proliferation in Hirschsprung disease by suppressing the expression of miR-1324 target PLCB1. *Cell Cycle* 17 1092–1101. 10.1080/15384101.2018.1480210 29895226 PMC6110588

