# Supplementary figures and images for: RNA-Seq Profiling of Circular RNAs During Development of Hindgut in Rat Embryos With Ethylenethiourea-Induced Anorectal Malformations
Source: Front Genet. 2021 Apr 13;12:605015. doi: 10.3389/fgene.2021.605015 (PMC8076906; doi:10.3389/fgene.2021.605015)

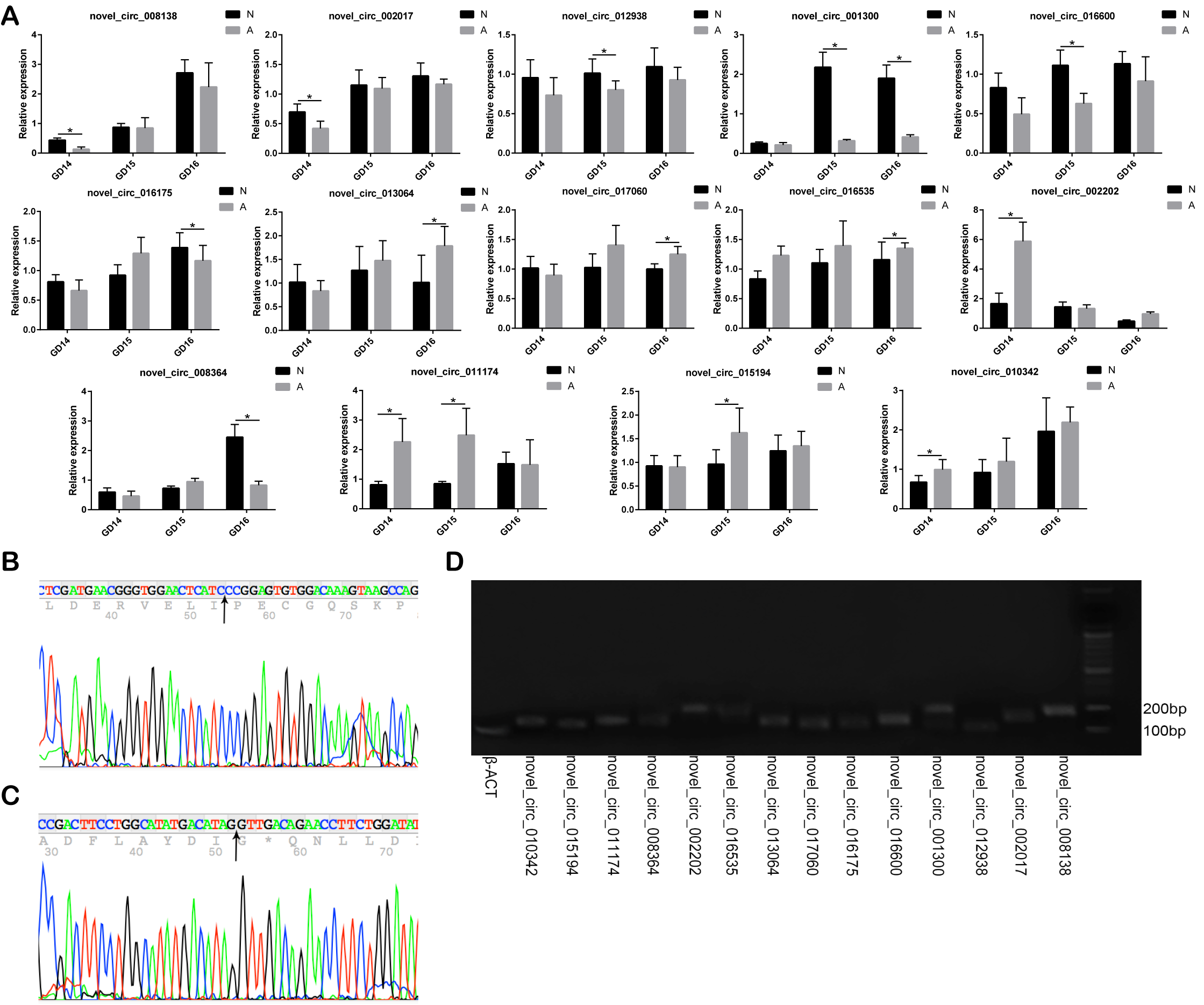

Supplement: Supplementary Figure 1 — (A) Fourteen circRNAs in rat embryonic hindgut tissues in the ARM group and normal group were validated by qRT-PCR. ∗p < 0.05, n = 5. (B,C) Two representative examples (novel_circ_002017 and novel_circ_008138) of qRT-PCR products confirmed by Sanger cloning and sequencing. (D) qRT-PCR products were visualized using agarose gels electrophoresis. GD, gestational day; A, ARM group; N, normal group. [file Image_1.TIF]
