# Supplementary material for: RNA-Seq Profiling of Circular RNAs During Development of Hindgut in Rat Embryos With Ethylenethiourea-Induced Anorectal Malformations
Source: Front Genet. 2021 Apr 13;12:605015. doi: 10.3389/fgene.2021.605015 (PMC8076906; doi:10.3389/fgene.2021.605015)
Supplement: Supplementary Table 1 — The sequences of the primers pairs. [file Table_1.docx]

|  | Forward (5′ - 3′ ) | Reverse (5′ - 3′ ) |
| --- | --- | --- |
| novel_circ_008138 | AAGTACTGATGGGGAGCGGC | GAGAAGGCTCGGACGTGAGG |
| novel_circ_002017 | TGAGTAACGGTGCCAAGTGC | CACTGCATTTGGGTAGCCTTCA |
| novel_circ_012938 | TCCTCCTCACTTGCCACCAC | ATGGCCAGGTAGAGGTGCTG |
| novel_circ_001300 | TGGTCCCATCAGACAAAGGCA | CGGTGTTGGTCCAGTACGGT |
| novel_circ_016600 | GCAATAGCGTCTCACATTGGCA | ACACTTGCTCTCCTGTGGCT |
| novel_circ_016175 | TCAGTGACCAACTCAGTGCCA | CTGGGAGAACCGTCGGTCAT |
| novel_circ_013064 | AAACCTCAGCCACAGCAGGA | GGGTGCTGGACAGTTTGAGC |
| novel_circ_017060 | CGGAGCCATCCTGAACTCCT | GCATTCGTTGTCGCCATTGC |
| novel_circ_016535 | TCCCGACCATCTGGCACTTC | CTGTAGATCTCATGCATAGGAGGACA |
| novel_circ_002202 | CAAGATCTTGGCCGCTGTGG | ACGCAACTGCTGAGACCCAT |
| novel_circ_008364 | ATCCACGCCAAGAGAGCCTC | TGACCTTCAGCTGCTGGACA |
| novel_circ_011174 | ATCAGCCCTGTCTCAACCGG | TCATGCCTGAGTGAGAAGCCT |
| novel_circ_015194 | TGAGGCCCTCAAAGCTCTGT | AACTTCCAGGCAGGGCACTT |
| novel_circ_010342 | AGACGGCGAATACTGGGTCG | TGGCCGCTTCATCTGCTCAA |
| β-actin | CCGCATCCTCTTCCTCCCT | GCCACAGGATTCCATACCCAG |

Supplementary Table 1. The sequences of the primers pairs.
